# Basic and clinical immunology – 3022. Inhibitory action of fexofenadine hydrochloride on mast cell activation *in vitro*

**DOI:** 10.1186/1939-4551-6-S1-P198

**Published:** 2013-04-23

**Authors:** Miyuki Suzuki, Atsuko Furuta, Kazuhito Asano, Harumi Suzaki

**Affiliations:** 1 Department of Otorhinolaryngology, School of Medicine, Showa University, Tokyo, Japan; 2 Physiology, Showa University, Japan

## Background

Allergic rhinitis is well known to be accompanied by inflammatory responses in the nasal wall and lumen including predominant infiltration of eosinophils, T cells and mast cells. With the discovery of IgE as the link between allergen exposure and mediator release, mast cells established their role as effector cells in the development and maintenance of allergic diseases. However, the influence of histamine H_1_-receptor antagonists on mast cells activation is not fully understood. The present study, therefore, was undertaken to examine the influence of histamine H_1_-receptor antagonist on mast cells activation by using an *in vitro* cell culture technique and fexofenadine hydrochloride (FEX).

## Methods

Spleen cells obtained from BALB/c mice were cultured for 3 weeks in the presence of FEX, and the number of mast cells was counted with alcian blue. We also examined the influence of FEX on mast cells activation. Cultured mast cells were sensitized with OVA-specific IgE and these sensitized cells were stimulated with OVA in the presence of FEX for 4 hours. The levels of tumor necrosis factor (TNF)-α, vascular endothelial growth factor (VEGF) and keratinocyte-derived chemokine (KC) were examined by ELISA.

## Results

FEX could not suppress mast cell growth from progenitor cells in spleen cell suspension, even when 500 ng/ml of the agent was added to cell cultures. On the other hand, treatment of mast cells with FEX caused suppression of factor production from mast cells by antigenic stimulation. The minimum concentration of the agent that caused significant suppression was 200 ng/ml.

## Conclusions

The present results strongly suggest that FEX exerts inhibitory effects on mast activation and results in favorable modification of clinical status of pollinosis patients.

